# Human Activity Recognition Using Inertial Sensors in a Smartphone: An Overview

**DOI:** 10.3390/s19143213

**Published:** 2019-07-21

**Authors:** Wesllen Sousa Lima, Eduardo Souto, Khalil El-Khatib, Roozbeh Jalali, Joao Gama

**Affiliations:** 1 Universidade Federal do Amazonas, Manaus 69080-900, Brazil; 2 University of Ontario Institute of Technology, Oshawa ON L1H 7K4, Canada; 3 Institute for Systems and Computer Engineering, Technology and Science—INESCTEC, Porto 4200-465, Portugal

**Keywords:** human activity recognition, smartphones, inertial sensors, features extraction

## Abstract

The ubiquity of smartphones and the growth of computing resources, such as connectivity, processing, portability, and power of sensing, have greatly changed people’s lives. Today, many smartphones contain a variety of powerful sensors, including motion, location, network, and direction sensors. Motion or inertial sensors (e.g., accelerometer), specifically, have been widely used to recognize users’ physical activities. This has opened doors for many different and interesting applications in several areas, such as health and transportation. In this perspective, this work provides a comprehensive, state of the art review of the current situation of human activity recognition (HAR) solutions in the context of inertial sensors in smartphones. This article begins by discussing the concepts of human activities along with the complete historical events, focused on smartphones, which shows the evolution of the area in the last two decades. Next, we present a detailed description of the HAR methodology, focusing on the presentation of the steps of HAR solutions in the context of inertial sensors. For each step, we cite the main references that use the best implementation practices suggested by the scientific community. Finally, we present the main results about HAR solutions from the perspective of the inertial sensors embedded in smartphones.

## 1. Introduction

Human activities have been commonly used to define human behavioral patterns. The availability of sensors in mobile platforms has enabled the development of a variety of practical applications for several areas of knowledge [1,2] such as:

- Health—through fall detection systems [3], elderly monitoring [4], and disease prevention [5].
- Internet of Things and Smart Cities—through solutions used to recognize and monitor domestic activities [6] and electrical energy saving [7].
- Security—through individual activity monitoring solutions [8], crowd anomaly detection [9], and object tracking [10].
- Transportation—through solutions related to vehicle [11,12] and pedestrian [13] navigation.

For this reason, the development of solutions that recognize human activities (HAR) through computational technologies and methods has been explored in recent years [11,14,15,16]. In this sense, the HAR problem has previously been treated as a typical pattern recognition problem, and more specifically, a classification problem, that is, to identify the activity being performed by an individual at a given moment. For this reason, most HAR solutions have been developed using artificial intelligence methods through various machine learning techniques, including shallow (e.g., Support Vector Machine (SVM), Decision Tree, Naive Bayes, and KNN) and deep algorithms (e.g., Convolutional Neural Network (CNN), Recurrent Neural Network (RNN), Restricted Boltzmann Machine (RBM), Stacked Autoencoder (SAE), Deeply-Connected Network (DFN), and Deep Belief Network (DBN)) [16,17,18,19].

The development of efficient solutions for HAR depends on understanding the concepts, limitations, and challenges. Human activities are defined as a set of actions that can be repeated over time in a given environment [20]. When these actions become noticeable and frequent, this set can be considered an activity, such as walking and cooking. However, the problem with the existing solutions that recognize human activities is related to the predictive capacity of the classification models adopted, since each individual tends to perform activities in different ways due to habits, personal preferences, and health. In addition, the number of activities performed by a human is much greater than current solutions are able to recognize. Even with such limitations, solutions developed in this area have presented interesting results in specific applications related to well-being through the recognition of users’ physical activities [19,21,22].

Smartphones have been commonly employed to develop HAR solutions because of the ubiquitous capability and diversity of sensors embedded in such devices. Smartphones are included in the scope of wearable computing [23,24], and these devices are considered part of mobile computing-based HAR systems. The advantage of smartphones over other wearable devices is associated with their ability to (a) capture and process data, (b) transmit and receive data, and (c) connect with other devices or sensors available in the physical environment. Inertial sensors such as the accelerometer and gyroscope are most commonly used to capture information related to acceleration and direction of movement of the human body, respectively. These sensors have allowed for the extraction of diverse information about the user that can be used to recognize individual physical activities [25].

HAR solutions based on smartphones with inertial sensors have evolved and followed a developmental methodology with well-defined steps such as data collection, segmentation and fusion, extraction and selection of features, and generation of classification models through machine learning algorithms [16,17]. Recently, the HAR area has converged to use new deep learning techniques that have changed the procedures commonly used for extraction and feature selection steps of traditional methodology [19]. These procedures refer to the way the features are extracted since the deep learning algorithms can automatically generate the features during training of the classification models, whereas in the traditional procedure, the features are defined manually.

This article analyzes the variations in HAR methodologies based on the recognition of users’ physical activities (e.g., walking and running) through smartphones equipped with inertial sensors. The article was motivated by the need for a comprehensive discussion about the main differences between the traditional methodology based on shallow machine learning algorithms and the methodology based on deep learning algorithms. Prior surveys approach the traditional methodology [11,14,16,18,22,26,27,28] and methodology based on deep learning [19,29] separately. In this sense, this article has three main contributions. The first contribution is an impact analysis of the extraction process for manual and automatic features using shallow and deep machine learning algorithms. The second contribution is the increment of the previous surveys along with the presentation of more complete information with topics related to the inertial sensors data fusion, impact of solutions on the battery power consumption in smartphones, and a variety of techniques to reduce data dimensionality.

The third contribution consists of a better presentation of the HAR area in the context of smartphones with inertial sensors. This presentation includes the description of users’ activities concepts, applications, and challenges. Furthermore, the following stages are described: (1) data understanding, (2) data preparation, (3) data modeling and inference, and (4) evaluation of HAR solutions. All of these steps are part of a well-defined methodology commonly used to develop HAR solutions. For each step of the methodology, we present a detailed description including a list of papers grouped by different forms of data processing. Besides, we highlight the best practice recommendations for implementing each step in future implementations. In addition, we present historical events to demonstrate the evolution of solutions, identifying the opportunities and motivating the development of new solutions for future research.

## 2. Understanding Human Activities

Human activities, such as bathing, cooking, working, and driving, can be defined as a set of actions performed by the user over a period in a given environment [20]. Formally, an instance or occurrence of an activity is defined by a sequence of sensor events. Events are defined as sequences of data formed by consecutive sensor readings in a given period. Each event, e, has the form e=(t,s,m) where t represents the time, s the sensor, and m the sensor message. In this way, a set of activities can be defined as $A=\left(a_{1},a_{2},\ldots ,a_{n}\right)$, where $a_{n}=\left(e_{1},e_{2},\ldots ,e_{k}\right)$ represents n-nth activity and $e_{k}$ the k-nth event [19].

Human activities can be categorized by the complexity level of the recognition process. Dernbach et al. [30] and Shoaib et al. [21], for example, categorize activities as simple and complex, while Khan et al. [31] and Reiss [32] categorize the activities as low-level and high-level. Both categorizations have the same meanings. Briefly, simple or low-level activities are those activities that can only be recognized by analyzing data from one or more sensors in a short period of time (e.g., walking and running). While complex or high-level activities can be seen as a set of low-level activities that can be recognized over a long period of time (e.g., work and shopping).

Most studies of HAR in smartphones focus on the use of inertial, acoustic, radio, and localization sensors. Based on this, activities on this scale can be divided into two groups [11,16]. The first deals with activities related to an individual’s movement (e.g., walking and running) and the second deals with activities related to an individual’s location (e.g., work and shopping). Studies related to movement activities focus on the analysis of users’ physical activities [25], while studies related to location activities focus on tracking users’ positions [13,33,34].

In this context, this survey focuses on detecting physical activities that are performed by users using inertial sensors (e.g., accelerometer and gyroscope) embedded in smartphones. The physical activities of the users are directly related to the movement and resting of the human body. Therefore, the activities detected in this context and those addressed by the solutions presented in this work include walking, running, lying down, standing, biking, driving, and climbing stairs. 

## 3. Research Method

To identify the papers presented in this article, we have used some systematic review techniques to maximize the amount of works in the HAR area for smartphones. For this, we get papers from the main digital libraries as IEEE Explorer, ACM, Springer, and Google Scholar. The query used to recover the works was based on the following research questions:

1. What are the methodologies used in HAR focused in smartphones instrumented with the inertial sensors?
2. What are the best practices, in terms of methods and techniques, for developing an efficient solution?
3. Which sensors can capture representative data capable of improving the assertiveness of the activity’s classification?

The query was composed for the combination of the keywords “recognition of human activity”, “smartphones”, “inertial sensors”, “machine learning”, and synonyms. For instance: 

(“human activity recognition” OR “activity recognition”) AND (smartphones OR “mobile phones”) AND (“inertial sensors” OR “accelerometer” OR “gyroscope”) AND (“machine learning” OR “classification algorithms” OR “deep learning”)

In addition to the automated search in the digital libraries, we also used the snowballing technique [35] give us more security in capturing relevant articles. Basically, we have analyzed the most important references cited by the retrieved works. The results are organized in the following sections.

## 4. General Motivation for the Smartphone-Based HAR Area

Why are smartphones one of the main tools used for recognizing human activities? This question can be answered by the fact that smartphones are portable and since they have computational power, communication capability, in addition to a variety of embedded sensors. These features have made smartphones a key ubiquitous platform for HAR due to their ability to extract and combine context information from different types of real-world environments. Lane et al. [15] cite four factors that demonstrate how a smartphone is an ideal platform for recognizing human activities. First, the smartphone is an inexpensive device that brings together various hardware and software sensors in one device. Second, smartphones are open and programmable devices. Third, smartphones have a high power of mass reach by distributing content and applications via virtual stores (e.g., app store). Finally, cloud computing allows developers to have extra features that serve as support and information sharing for these devices. In addition, data on users’ activities, preferences, and social interactions can be used to recognize, monitor, and react to virtually any phenomenon, be it physical, psychological or social.

To reinforce the motivation to use smartphone device in the HAR area, the main historical events that marked the evolution of the HAR area from the perspective of smartphones are as follows.

One of the first historical milestone was in 2006, when the first HAR solutions appeared that explicitly used smartphones [36,37]. At that time, the first studies were performed using data analysis extracted from the GSM sensors and accelerometer for monitoring users’ mobility. During this period, all data processing was performed on a computer (offline processing) because smartphones had limited computational resources.

As of 2007, the literature has advanced to the development of the first collaborative solutions [38,39,40]. The communication and processing model adopted by these solutions adheres to the following flow: smartphone data was collected, sent to a server on the Internet, where the users’ information was shared. In general, such information was used to improve the accuracy of the classification models of machine learning algorithms. Only until 2008, with the evolution of processing and storage technologies in smartphones, did solutions begin to appear in which the data collection and processing were executed in the smartphone itself [41].

As of 2009, smartphones were equipped with an even greater number of sensors, allowing users to recognize new activities, such as human voice recognition. The works of SoundSense [42] represent this scenario very well with recognition of activities based on the ambient sound, such as group conversations. Around the same time, more studies focused on the development of applications in the healthcare area, as chronic diseases detection based on the locomotion problems of the users [4,43,44].

As of 2010, researchers focused on improving the specificities of the HAR recognition process, such as (i) efficient data collection through continuous sensing to improve the energy efficiency of batteries [45], (ii) improvement of classification models using a set of classifiers [46], and (iii) detection of transition intervals between activities [47]. In addition, Berchtold et al. [39] proposed the creation of a HAR cloud service that allows for constant updating of the classification models embedded in smartphones through feedback from users.

Starting in 2011, more specific solutions emerged, such as the first discussions related to the effects of location and orientation of the smartphones on the users’ body [25,48]. For example, Henpraserttae et al. [48] showed that the smartphone located in the hand and on the waist of an individual produces different signals and, consequently, require different analyzes to recognize the same activity. During the same time, the first public database was published, as the WISDM database [49]. Public databases assist in the validation and comparison of new HAR methods over existing ones.

In 2012, the first studies related to the recognition of more complex activities using smartphones were published. Dernbach et al. [30] and Khan et al. [31], for example, combined data from the inertial sensors to recognize daily (e.g., cooking) and physical activities. Das et al. [50] combined sensor data from smartphones and smart environment sensors to recognize users’ daily activities. Other works focused on improving online solutions so that the entire data process occurs in the mobile device [46,47].

From 2013, data fusion techniques for multiple sensors were applied in the feature extraction step [51]. In addition, more detailed studies for discovering accurate lightweight features were performed [52]. In 2014, some research focused on the data segmentation step with the objective of measuring the impact of time window size on the accuracy of classification models [53]. From 2015, the HAR area began to converge with the application of deep learning classification algorithms, with the first work developed by Alsheikh et al. [54]. From there, the classification models generated by the deep learning methods became the state of the art of classifying activities.

In 2016, new HAR researchers emerged focusing on the data streaming area [55]. At this point, such problem has been treated as an online learning problem in order to minimize the need to store a historically required training of the classification models. In addition, this method is based on the novel detection with the objective of mapping new activities that, by chance, are not represented in the classification models. The method also includes continuous interaction with the user through active learning. In addition, new studies [56] related to transition-between-activity recognition (e.g., sit-to-stand) emerged in an attempt to eliminate classification errors of the models, since the data referring to transitions are considered noise in the database.

In 2017, several studies [31,57,58,59,60] performed comparative analyzes among the various HAR solutions available in the literature. Most of the analyzes tried to discover the effectiveness of the features in the classification models generated by machine learning algorithms. Recently, in 2018, frameworks have emerged to encapsulate all the previously studied steps and methods into a generic architecture [61]. From there, new implementations of HAR in API’s (Application Programming Interface) format could arise to facilitate the development of HAR applications.

## 5. Human Activity Recognition Process

The process of human activities recognition is very similar to a general-purpose pattern recognition system and corresponds to a set of steps ranging from data collection to activities classification. This process involves a set of transformations of the raw data extracted from sensors to generate efficient classification models of human activities. The HAR methodology for smartphones equipped with inertial sensors can be divided into two approaches based on machine learning techniques as shallow algorithms (e.g., SVM, KNN, and decision tree) and deep algorithms (e.g., CNN, RNN, RBM, SAE, DFN, and DBM). The main difference between these approaches is the way in which the features are extracted, that is, whether it is manually or automatically extracted [19].

This difference is highlighted mainly because the conventional process of feature extraction is limited by human knowledge [62]. In the case of data collected from inertial sensors, the features are commonly extracted based on two main domain features: time domain and frequency domain [63]. The disadvantage of this conventional approach is that, in some cases, human expertise may not always be able to select the best set of features for different scenarios. Another disadvantage is that this approach can generate irrelevant features, making it necessary to apply methods that reduce the dimensionality of the data, such as feature selection, since unnecessary features can affect the performance of classification algorithms.

In order to overcome these disadvantages, deep learning algorithms provide a benefit to the feature extraction step due to their ability to automatically generate features. These algorithms are capable of generating complex and high-level features that represent the data well and generate efficient classification models for different scenarios. For this reason, deep learning methods are considered the state of the art in areas such as computational vision and natural language processing [64].

**Figure 1:**
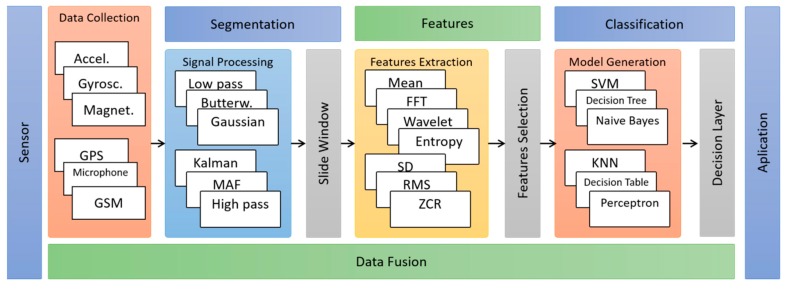
Set of steps based on the manual features’ extraction used by shallow learning algorithms.

**Figure 2:**
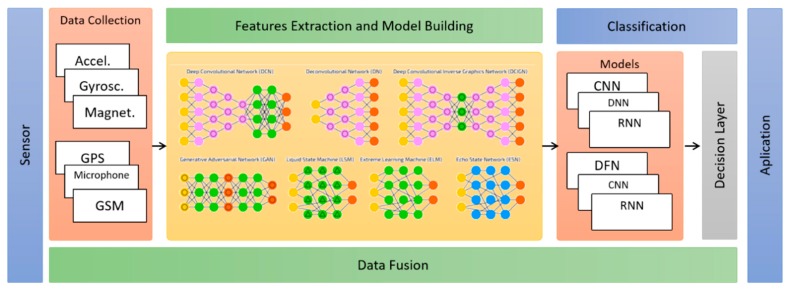
Set of steps based on the automatic features’ extraction used by deep learning algorithms.

To illustrate the difference between the two approaches, Figure 1 shows the steps commonly used in conventional approaches, highlighting the segmentation and features extraction steps. Figure 2 shows the steps commonly used in the deep learning approach, where the features are implicitly generated in the hidden layers of the neural networks, during the training and construction phase of the classification models. The other steps are shared between the two approaches. Section 5.3 contains more details of the feature extraction step for both approaches.

The segmentation step is part of the data preparation process, in which data is divided into segments known as time windows. Time windows are used in the process of extracting features in the conventional approach. The deep learning approach does not need to use time windows because the data processing occurs directly in raw data. On the other hand, some works [25,46,65] add an earlier step regarding the pre-processing of raw data in order to minimize noise caused by anomalies related to environmental conditions, movements, and changes in user behavior during data collection. The most commonly used noise elimination techniques are Lowpass filters [65], moving average filter [25] and Kalman [46]. In this case, both approaches can use this strategy.

The initial step in data collection studies is as follows: raw data is collected from smartphone sensors, such as accelerometer and gyroscope. For this, a set of parameters such as type, time, and frequency of data collection, as well as, the position and orientation of the smartphone on the user’s body should be taken into consideration. Smartphones commonly used in data collection have embedded operating systems like Android, IOS, and Symbian. The last step concerns the construction of classification models to infer human activities. Classification models are generated based on shallow or deep machine learning algorithms.

The data fusion step is a cross-process used to combine data from multiple sources. This cross-sectional aspect occurs because the data can be combined in any of the steps described above. For example, (i) in the data collection and segmentation steps where signal fusion techniques such as Magnitude can be applied, (ii) in the feature extraction step where the strategy of concatenating vectors of features can be applied, and finally (iii) in the classification step where an ensemble of classifiers can be used to aid in the inference decision. All these strategies aim to enhance the accuracy of activities classification. Section 5.3 contains more details about data fusion strategies.

### 5.1. Data Collection

In general, data collected from smartphone inertial sensors are arranged chronologically in the form of a time series. Accelerometer data, for example, are represented by a set of three vectors $acc_{i}=\left(x_{i},y_{i},z_{i}\right)$, where i=(1,2,3,…,n). The accelerometer is the most commonly used sensor in the HAR for smartphones because it thoroughly represents the movement activities of users. 

In order to generate good classification models, the following elements must be considered: type, time, frequency, position, and orientation of the smartphone with the user’s body. Lockhart and Weiss [66] proposed two types of data collection based on the level of naturalness with which the data is collected:

1. Natural: Users perform their daily activities normally without intervention in their behavior by the application.
2. Semi-natural: Users perform their daily activities normally, but the user is required to perform the activities from the experiments at least once, that is, the user must ensure that all activities related to the study have been performed.
3. Laboratory: Users perform activities systematically in a controlled environment with previously defined steps.

The literature shows that data collection performed in laboratories tends to generate more accurate classification models because the activities in this type of data collection are previously defined. However, the models generated with these kinds of datasets lose accuracy when applied in real contexts due to the diversity of users’ behavior. On the other hand, models generated with natural datasets tend to be more generic and can be applied to groups of people with similar behaviors. Table 1 presents some works divided by the type of data collection.

The frequency rate of data collection is also an important factor that should be considered during data collection since the frequency rate contains relevant information about the movement of the human body [81]. Theoretically, the frequency rate represents the amount of sample data collected every second (Hertz).

In the literature, the frequencies used in the extraction of cellular sensor data vary from 1 Hz to 200 Hz for the inertial sensors. To find out the ideal frequency, Khusainov et al. [18] proved through experiments that the frequency of 20 Hz contains enough information about human physical movements. Table 2 shows a list of papers that have conducted studies on several frequency bands.

The position of the smartphone on the user’s body is another factor that greatly influences the quality of the data collected and the accuracy of the classification models. For example, data collected with the smartphone positioned at a user’s waist produces different signal patterns from a smartphone placed in a user’s hand [44,49]. Table 3 shows a list of works separated by smartphone positions on the user’s body.

Generating generic models for the activities’ recognition using smartphones located at different positions on the user’s body is still a challenge to be overcome. Studies performed with the smartphone at different positions show that the waist is the best position to recognize physical activities since human body movements originate from this region [48]. Moreover, some works described in [81] have employed solutions that are independent from the position of the smartphone on the human body. In brief, these solutions have focused on data extraction with the smartphone located in all relevant positions on the user’s body.

The orientation of the smartphone (e.g., portrait and landscape) is also another factor that influences the accuracy of the classification models. What makes the classification models dependent or independent of orientation are the types of features used in the training phase. For example, the features signal magnitude is considered an independent orientation feature, since their values do not change with device orientation changes. Table 4 presents a list of works that observed the orientation of smartphones to generate classification models and Section 5.4 presents details about these features.

Finally, the diversity of the data allows for greater generalization of classification models. The literature review shows that the number of individuals did not exceed 49, as shown in the datasets presented in Section 8. This occurs because of the difficulty in persuading users to provide their personal information. To diversify the scenarios, it is necessary to obtain data from people of different ages and groups, and with different levels of health and locomotion, among other factors.

### 5.2. Segmentation

Segmentation is intended to separate data into meaningful sub-groups that share the same characteristics. In the context of the inertial sensors, the data subgroups are represented by signal segments in a given time interval. The objective is for each segment to contain sufficient characteristics that allow the recognition of a human activity at a given moment, that is, the data analysis must be done exactly during an execution time interval of each activity.

To achieve this goal, the data is divided into consecutive segments so that each of them is analyzed separately and sequentially. This process is known as time windows (or sliding windows). Sliding window-based segmentation is often used to manipulate data from inertial and audio sensors because events are represented by continuous values. This approach divides sensor events into organized subsequences over time.

Cook and Krishnan [20] define a sliding window as derived from a sequence of events $X=\left\{x_{1},x_{2},\ldots ,x_{n}\right\}$, where x represents the value and n nth value of the sequence. The time window is represented by a subsequence $X^{\prime }=\left\{x_{p},x_{p+1},\ldots ,x_{p+w-1}\right\}$, where w represents the size of the time window and p represents an arbitrary position, such as 1≤p≤n−w+1, where n represents the size of the sequence. In the case of inertial sensors, the data is represented in a three-dimensional plane along the axes $x_{i},y_{i},z_{i}$, where i=(1,2,3,…,n).

Time-window based segmentation can be manipulated in two ways [18]: Overlapping and non-overlapping windows. Non-overlapping windows are segments in which their values do not intersect with the values of other windows, i.e., $X_{1}\cap X_{2}=\emptyset$. Overlapped windows are segments represented by a percentage that defines how many samples from the previous window intersect the samples from the next window, i.e., $X_{1}\cap X_{2}\neq \emptyset$. For example, given a time window with 100 samples, 50% overlap means that 50 samples from the previous window will be part of the sample set from the next window.

In the context of inertial sensors, window sizes are measured based on the time interval and frequency rate of data collection. In addition, the windows may have fixed or variable sizes. The number of fixed size samples is defined based on the time commonly measured in seconds. Studies show that the ideal size for fixed windows varies around 2 to 5 s considering a frequency of 20 Hz to 50 Hz [21,53,58]. On the other hand, the number of samples of the variable length windows can be defined according to changes in the mean and variance of the signal, for example. However, to the best of our knowledge, there have been no studies with variable window sizes. All solutions found so far use fixed-size time windows. Table 5 shows a list of works with their respective time window sizes used.

For studies related to variable sizes of time windows, a recommended technique, that detects changes in the signal mean, is the Page Hinkley technique proposed by Sebastião et al. [84]. The intuition behind this idea is that the boundaries of the time windows may be exactly the changes in the signal mean over time. This solves the problem related to mixed data from two or more activities in the same time window.

Another important factor that influences the segmentation process is the data cleaning through the noise reduction of the inertial sensors. Missing values, incorrect values or outliers can characterize such noises. Noise can be eliminated using specific techniques commonly used in the area of signal processing. A state-of-the-art survey shows that smartphone-based HAR solutions primarily use low-pass [4,31,51,52,77,78] Butterworth [65], Kalman [46], and Moving Average [25,52] filters.

### 5.3. What Are Features?

Intuitively, a feature can be defined as an element endowed with some useful information about the data which it represents. In the context of HAR, this concept can be used to represent the different movement patterns of users’ physical activities. For example, the “run” activity requires greater effort from the human body to generate movement compared to “walking” activity. Therefore, the intensity of the effort of each one of them is transferred to the inertial sensors, directly influencing the data distribution collected from these sensors. Consequently, we can find ways to highlight the difference between “walking” and “running” activity using, for example, statistical data information. Thus, the mean and variance of the data may be useful to highlight the difference between these two activities.

In this context, the literature classifies the features in different domains of representation. Each domain has a set of specific formulas that extract different useful information from the inertial sensors signals. The classification groups of feature domains defined in the literature are [63]: time domain, frequency domain, and discrete domain. The time domain has mathematical functions used to extract statistical information from the signals. The frequency domain has mathematical functions that capture repetitive patterns of signals and are often related to the natural periodicity of the activities. The discrete domain uses symbolic representation techniques to represent signal patterns through discrete information. The discrete domain features are rarely exploited in the HAR area. In fact, Siirtola et al. [71] and Figo et al. [63] use the SAX discretization technique for extraction of features. Therefore, such domain is not the focus of this research.

#### 5.3.1. Time Domain

This section presents details about the most commonly used time-domain-based characteristics in the context of smartphone inertial sensors. These features can be divided into two types of functions: statistical functions and non-statistical functions. The statistical functions involve calculations such as minimum, maximum, average, standard deviation, among other formulas. The non-statistical functions involve several calculations such as areas, and calculation of Bins Distribution, among others. Table 6 shows the set of time domain features as found in the literature. All of them are applied to the x, y, and z axes of the inertial sensors.

Among the features mentioned above, some special features can generate other new features through a process of chaining mathematical functions. For example, the signal magnitude feature can be combined with other features, such as mean and variance, and generate new features from this combination [73]. The same happens with the features based on the vertical and horizontal components of the signals [48,79]. In addition, the signals generated by these special features present sizes equal to the size of the original signals, while the other normal features generate compressed signals with sizes equal to the number of time windows defined in the segmentation step. In other words, these features work as data fusion techniques, since the coordinates x, y and z are transformed into only one axe. For these reasons, we classify the features with this type of behavior as low-level features, where the extraction process is performed in the raw data.

Signal magnitude is an orientation independent feature extracted from multiple-dimensional sensor data. According to Khusainov et al. [18], the purpose of magnitude is to assess the degree of movement intensity based on thresholds derived from acceleration peaks. This is possible because the magnitude highlights the variation of signals caused by the merging of values between different coordinates of the inertial sensors. (1) Shows how to calculate the signal magnitude in data extracted from inertial sensors whose coordinates are x, y and z:

(1) $$M\left(X\right)=\sqrt{x^{2}{}_{i}+\;y^{2}{}_{i}+\;z^{2}{}_{i}}$$

Likewise, the features based on the vertical and horizontal components are also considered independent orientation features [25,72]. These features also fuse the x, y and z coordinate values of the inertial sensors. Formally, (2) presents the formula for the calculation of the vertical component:

(2) $$v_{i}\;=\vec{a_{i}}.\;\hat{g}$$

where $\vec{a_{i}}=(x_{i},y_{i},z_{i}),\;1\leq i\leq m$, i represents the values of the samples in a window of size m. The value of $\hat{g}$ is a unit vector representing the gravity contained in the signal. The gravity $\hat{g}$ can be extracted according to (3):

(3) $$\hat{g}=\;\frac{\left(\bar{x},\bar{y},\bar{z}\right)}{\left\|(\bar{x},\bar{y},\bar{z})\right\|}\|$$

where $\bar{x},\bar{y},\bar{z}$ represents the average of the values of each coordinate contained in a time window and $\left\|\left(\bar{x},\bar{y},\bar{z}\right)\right\|$ represents the vector norm of coordinate values. The features based on the horizontal components ($h_{i}$) are derived from features based on the vertical components $\left(v_{i}\right)$. Thus, the horizontal component is calculated by the formula in (4):

(4) $$h_{i}\;=\;\|\vec{a_{i}}-v^{proj}{}_{k}\|$$

where, $v^{proj}{}_{k}$ means a vertical component projection calculated by the scalar product of the vertical component and signal gravity (5).

(5) $$v^{proj}{}_{k}=v_{i}\hat{g}$$

In addition, the magnitude features and vertical and horizontal components are considered independent of smartphone orientation on the user’s body

#### 5.3.2. Frequency Domain

This section presents details about the most frequently used frequency domain features in the context of smartphone inertial sensors. These features present an alternative to signal analysis based on the frequency spectrum of the values of a certain time window. The features of Table 7 are calculated based on the low-level fast Fourier transform (FFT) or Wavelet features. Table 7 shows the set of frequency features found in the literature. All of them are applied to the x, y, and z axes of the inertial sensors.

The frequency domain features described in Table 7 depend strictly on the low-level Fourier and Wavelet transformed features. Both transformations consist of a mathematical tool that transitions between variables over time for frequency variables, that is, the signal is decomposed into a set of real and imaginary values that represent components of waves called frequencies.

In the context of HAR, the transformations are useful for representing repetitive patterns of signals in terms of frequency. In order to calculate the Fourier transformation, an efficient algorithm is used to calculate the discrete Fourier transform (DFT), called fast Fourier transform (FFT), whose formula is represented by (6):

(6) $$FFT\left(X\right)=\sum_{k=0}^{n-1}x_{k}e^{-2\pi ij\frac{k}{n}}$$

where $x_{k}$ is a sequence of size n which represents a contiguous signal and $e^{-2\pi ij\frac{k}{n}}$ represents the nth primitive root of each unit of $x_{k}$. More details about the primitive root can be found in [84]. The calculation of the Wavelet transformation is similar to the calculation of the Fourier transform, the difference being that the values of a range are represented in terms of orthogonal bases. There are several ways to calculate the Wavelet transform by observing continuous and discrete values. However, in the context of HAR, the literature uses a simplified form called Haar Wavelet. This form is represented by the basic Wavelets calculation formula presented by Strang [85].

In addition, some features belonging to the time domain can be adapted to the frequency domain, such as peak frequency amplitude of coefficients, coefficient mean, coefficient area, among others, since real and imaginary coefficients also form value vectors. You can then extract statistical information about them. The features derived from the Fourier and Wavelet transformations are dependent on the orientation of the smartphone on the user’s body.

### 5.4. Feature Extraction

In general, the feature extraction corresponds to a process of data transformation performed on the segmented data. In the context of inertial sensors, this process is necessary because the raw data, represented by the signal, are not suitable for use by conventional machine learning algorithms [20,86]. This section presents details about how the features of time and frequency domains are extracted from the signal. In addition, this section addresses aspects related to data dimensionality reduction in the datasets generated after the feature extraction step.

#### 5.4.1. Time and Frequency Domain

Time and frequency domain features are extracted in the same manner, i.e., both are derived from processes performed on the time windows defined in the segmentation step. For this reason, the size and overlap rate of time windows directly implies the quality of the features. The feature extraction process generates a new dataset used in for training and the generation of activities classification models. Table 8 shows the studies distribution separated by the feature domains.

Some important data indicates that most HAR-based work on smartphones with inertial sensors uses the time-domain-based features [52,66]. The reason for the wide use of this approach is that, in general, the time domain features have a lower computational cost when compared to the frequency domain features [74,81]. In contrast, the features of the frequency domain can better represent context information in terms of signal patterns.

Low-level features described in Section 5.3 can be divided based on the smartphone orientation on the user’s body (landscape and portrait), that is, the features can be dependent or independent from the device orientation on the user’s body. The magnitude feature, for example, is considered an independent orientation, whereas the FFT and Wavelet features are considered orientation dependent. As a result, all new features derived from any low-level feature inherit the dependency or independency orientation. On the other hand, when applied in isolation, the time and frequency domain features are all orientation dependent.

#### 5.4.2. Data Dimensionality Reduction

Dimensionality is attributed to the number of features contained in a dataset, where each feature represents a dimension in space. In this way, a large space of features evidences two problems. The first is related to the cost of data processing and the second to the accuracy of the classification models generated in the learning phase. In addition, there is the problem of dimensionality curse that can provide high error rates in a classifier [87]. In this sense, the process for data dimensionality reduction involves removing irrelevant features to improve the accuracy of classification models. For this reason, Khusainov et al. [18] affirm that the choice of features is more important than the choice of classification algorithms since the poor quality of the features can negatively impact the accuracy of any model generated by the conventional machine learning algorithms.

The literature about data dimensionality reduction is quite extensive [87], however, few studies have used such techniques in the context of smartphone-based HAR. Even so, it is possible to identify two types of techniques used in this context. The first deals with techniques that act after the feature extraction step and the second deals with techniques that act during the feature extraction step.

The first type is characterized by feature selection techniques. These sets of techniques select the most representative features from all features available in the dataset. In the context of HAR, we can mention some examples used in the solutions proposed by Khusainov et al. [18] and Khan (2011). These methods were used based on the analysis of the information gain of the features (Info-gain method) and how each one is correlated with one another (Correlation-based Feature Selection method). Both methods analyze the impact that each feature has on the performance of the classification models, a feature that does not have information gain or is highly correlated with each other can be discarded from the dataset.

While the features selection techniques define the subset of features that best discriminate human activities, the methods that operate during the feature extraction step combine the features to reduce the data dimensionality. Besides, new features that have lower intra-class variance and higher inter-class variance are generated to increase the separability of activities before feeding the classifier [88]. In this context, the techniques most used in the context of HAR are Principal Component Analysis (PCA), Linear Discriminant Analysis (LDA), and Kernel Discriminant Analysis (KDA).

Khan et al. [31] present the results of a series of experiments related to the data dimensionality reduction in the context of the inertial sensors. The results show that the methods that work during the feature extraction process are more efficient compared to the features selection methods. Among them, the feature generated by the KDA obtained the best classification models. In another paper, Khan et al. [52] combined the KDA with several sets of features, including time and frequency domain. Thus, the combination of the KDA and time domain features obtained better results in the accuracy of the classification models.

Recently, new dimensionality reduction methods have been employed in the context of HAR. For example, Hassan et al. (2017) use the Kernel PCA (KPCA) method with a statistical kernel that improves PCA performance. Siddiqi et al. (2014) use the nonlinear method called Stepwise Linear Discriminant Analysis (SWLDA) that selects discriminant features using regression methods combined with statistical technique F-test. Finally, Guo and Wang (2018) used a modification of the LDA method called Robust Linear Discriminant Analysis (RLDA).

#### 5.4.3. Feature Extraction based on Deep Learning

Features extraction methods based on deep learning techniques act differently from conventional feature extraction methods. The main difference between the two approaches is that the deep learning algorithms are able to generate the features automatically during the training process of the classification models. Such models are trained with more complex features and, in some cases, belong to unknown domains. In addition, features may change from one database to another, even if users perform the same activities. This is because neural networks can adapt to the distribution of data. For this reason, features extracted through deep learning methods have generated the best classification models in the HAR context, since, unlike traditional methods, deep learning methods are capable of designing significant and high-level features tailored to each scenario and data type [19].

To better understand the feature extraction process based on deep learning, it is important to know the basic structure of deep learning algorithms. In summary, the deep learning methods are based on neural networks with multiple layers that depend each other [89]. Each layer represents a level of the problem abstraction, i.e., the greater the number of layers, more details of the problem are mapped to the classification models. For example, in the context of image processing, the first layer may contain features that represent the image texture, while the second layer may contain other features that represent the lines and edges of the image. The same analogy can be made in the context of smartphone-based HARs. In this way, the first layer can represent the intensity of the movements of each activity and the second layer can represent the correlation between the movements. Thus, each layer of a deep neural network can represent a set of features referring to a level of detail of a given problem.

There are several deep learning methods described in [89]. However, in the context of HAR for smartphones only five methods were identified, of which they are: Deeply-connected network (DFN), Convolutional Neural Network (CNN), Recurrent Neural Network (RNN), Long Short-Term Memory (LSTM), Stacked Autoencoder (SAE), and Restricted Boltzmann Machine (RBM). If we only analyze from the perspective of feature extraction, all these methods are similar, with differences in the number of layers and in the way the layers are connected to each other.

A CNN is represented by successive convolutions and poolings between the layers. The convolution is a mathematical tool used to treat a matrix by means of another kernel matrix. The result is a linear transformation of all elements of the original matrix. In practice, this transformation causes effects such as enhancing the edges of an image or the type of motion of an activity.

The role of pooling in this context is to resize the matrix so that spatial size is reduced to reduce the number of parameters and operations in the network. In practice, this means that a 5 × 5 size matrix can be reduced to 2 × 2. In the context of a CNN, the features are represented by the neurons of the subsequent successive layers represented by convolutions and poolings. The overview of the other methods is simpler and limited to the number of neurons and hidden layers of neural networks.

In terms of data input, each coordinate represents an input channel for the neural network. In this way, the processing is done by successive 1D convolutions. Otherwise, Wang et al. (2017) presented a data pre-processing where the three-dimensional signal is transformed into an image and, thus, the processing in the neural network is done through 2D convolutions.

The DFN method is characterized by a denser traditional Artificial Neural Network (ANN), i.e., the DFN contains many hidden layers (deep) in contrast to the traditional ANN that only has a few shallow layers. The SAE method is characterized by the use of a stack of autoencoders. Autoencoders are neural networks where hidden layers learn the features in an unsupervised way (pre-training) through a process of encoding and decoding data entry. The RNN method is characterized by a recurrent neural network that uses temporal dependencies between the neurons. Likewise, the LSTM acts in the temporal context based on memory units, where it is possible to “forget” or “update” some information from the hidden layers when necessary. Finally, the RBM method is characterized by a bipartite neural network, without direction and with the neurons of the layers completely connected to each other. A stack of RBMs is called the Deep Belief Network (DBN).

In addition, some papers use a combination of deep models. For example, Ordónez and Roggen [90] and Yao et al. [91] present examples of how to combine CNN and RNN. More details on each of these methods can be found in [89]. The number of features generated by any of the methods mentioned above depends on the definition of training hyperparameters of the neural networks. 

The main parameters used in the configuration of a neural network are a number of layers, number of neurons for each layer, number of times, learning rate, regularization weight, and activation function [60].

Although deep neural networks are able to generate the features automatically, Hassan et al. (2017) verified how these networks behave with the conventional features of time and frequency domains. In this case, the experiments were performed with 561 features using a DBN method architecture. The results presented better accuracy compared to conventional methods with an average difference of around 2%. Furthermore, prior to targeting the HAR area for deep learning, Khan (2011) realized, through experiments, that a hidden neural network with several hidden layers was able to generate useful features without resorting to the features of time and frequency domain.

### 5.5. Training and Classification

After the data processing in the segmentation and features extraction steps, the next step is to use classification algorithms that are responsible for generating classification models to infer human activities. In this context, the classification algorithms are divided into two groups. The first deals with conventional machine learning algorithms and the second deals with deep learning algorithms. In this way, the inference (or classification) models are generated through a training process of the classification algorithms. These models are generated from a training dataset where the activity samples must be properly labeled with the activities. Formally, the training process of these models is defined as follows [20]:

Given a random variable X belonging to an n-dimensional plane, this variable corresponds to attributes, or features, extracted from the sequences of sensor events. Thus, $X=\;\langle x^{1},x^{2},\ldots ,x^{n}\rangle$, where x represents a feature and n the number of features. The variable X has an associated y variable that represents the target attribute or class of the variable X. Thus, $y=\;\langle y^{1},y^{2},\ldots ,y^{n}\rangle$,where y represents the class and n the number of classes. Therefore, the set of training L consists of pairs $\left\{\left(x^{1},y^{1}\right),\left(x^{2},y^{2}\right),\ldots ,\left(x^{n},y^{n}\right)\right\}$, where $\left(x^{n},y^{n}\right)$ represents an instance formed by a set of attributes $x^{n}$ and a class $y^{n}$. The classification models are categorized as follows [22,92]:

1. Impersonal or generic: Models are trained with data from one user group and tested on another group of different users.
2. Personal or specific: Models are trained with data from only one user and tested with the same user.
3. Mixed: models are trained using the entire database without distinction between users.

In addition, classification models can be generated based on three strategies:

1. Cross-validation: the database is randomly divided into 10 equal parts, where the models are generated with 9 parts and tested with the remaining part. This is repeated until all parts are individually used as training. The final accuracy consists of the average of the 10 classification models generated in 10 training rounds.
2. Leave-one-subject-out: This strategy is similar to cross-validation, but instead of being randomly divided into equal parts, the data is divided by the user. The data of each user is used as a test.
3. Leave-30%-out: This strategy consists of dividing the data into 70% for training and 30% for testing.

In general, previous studies have sought to develop impersonal models with high accuracy rates; however, generating recognizers of human activities that consider factors such as age and health of the user is still a challenge. One solution to this problem would be to generate classification models for each user profile, such as children, adults, people with locomotion difficulties, among others.

The shallow machine learning algorithms commonly used to recognize users’ physical activities are represented by Naïve Bayes, Support Vector Machine (SVM), neural networks, KNN, and the decision tree family algorithms, for example. These algorithms use the time and frequency domains feature in the training process of the classification models. Table 9 presents a list of papers that use these methods. This article does not attempt to provide theoretical information about how each of these algorithms work, more details about each of them can be found in [20].

Likewise, deep learning algorithms have recently been used to recognize users’ physical activities on smartphones. In this context, the most widely used classification algorithms are a Deep-connected network (DFN), Convolutional Neural Network (CNN), Recurrent neural network (RNN), Long Short-Term Memory (LSTM), Stacked Autoencoder and Boltzmann machine (RBM) described in the Section 5.4.3. Table 10 presents a list of papers that use these methods. This article does not attempt to provide theoretical information about how each of these algorithms work, more details about each of them can be found in [64].

The main reason for using deep learning techniques is due to the success in the areas of image processing and natural language. From the perspective of HAR, these algorithms have generated good classification models, and are currently considered the state of the art in the HAR area. Therefore, our discussion addresses the main reasons that led to the migration from the HAR area based on smartphones to the use of the deep learning methods, as well as the advantages and disadvantages pointed out by each approach.

#### Evaluation Metrics

The performance of a particular classification model is evaluated by a set of metrics that inform, in mathematical terms, how reliable the model is in the HAR process. The key evaluation metrics commonly used in the smartphone-based HAR literature are [20]: accuracy, sensitivity, specificity, precision, recall, and f-measure.

Accuracy is the most common metric used to evaluate classification models. In the context of HAR, accuracy is calculated by dividing the number of correctly classified activities, c, and the total number of activities n. The formula of accuracy is shown by (9).

(7) Accuracy =c/n

Accuracy gives a general idea of classification models. However, this metric treats the classes as equally important in a dataset. This leads to accuracy being an inefficient metric in unbalanced databases. To solve this problem there are other metrics that evaluate classes separately, such as sensitivity and specificity. Sensitivity analyzes the True Positive (False Negative) rate for each class. The formula presented in (10) shows how the sensitivity is calculated:

(8) $$sensitivity=\frac{VP}{VP+FN}$$

where VP means true positives and FN means false negatives. In contrast, specificity analyzes the True Negative (False Positive) rate for each class. The formula presented in (11) shows how specificity is calculated:

(9) $$specificity=\frac{VN}{VN+FP}$$

where VN means true negatives and FP means false positives. It is similar with accuracy, recall, and f-measure metrics. Accuracy analyzes the hit rate from true positives to false positives, while recall reviews the rate from true positives to false negatives. The formulas in (12) and (13) show how to calculate the precision and recall metric.

(10) $$\mathrm{precision}\;=\frac{VP}{VP+FP}$$

(11) $$\mathrm{recall}\;=\frac{VP}{VP+FN}$$

The metric f-measure deals with a score resulting from the combination of precision and recall values. The idea of this metric is to provide a generic value that represents these two metrics. The formula of (14) shows how f-measure is calculated:

(12) $$f-measure=\frac{{\left(1+\;\beta \right)}^{2}*\;revocacao\;*\;precisao}{\beta^{2}*\left(revocacao\;+\;precisao\right)}$$

where β is a weight coefficient that is commonly assigned value 1. Previously, only accuracy has been used to measure the performance of HAR models.

## 6. Data Fusion

The data fusion step corresponds to the process of integrating multiple data and/or knowledge that represents a real-world object in a consistent, precise, and useful way [133]. Data fusion techniques are used to improve data consistency and assist in the extraction of increasingly complete information so that a given knowledge is reinforced by the data aggregation of multiple sources.

Saeedi [13] and Tsinganos and Skodras [134] present three levels at which data fusion techniques can be applied. The first level occurs directly in the raw data during the segmentation step. The second level occurs in the feature extraction step by concatenating feature vectors from multiple sources. Finally, the third level occurs in the decision layer by combining the results of several classifiers. A close look at the HAR literature for smartphones shows that data fusion techniques are more commonly applied in the context of the second and third level. In this perspective, Vaizman et al. [135] and Köping et al. [61] present three data fusion strategies in the context of HAR for smartphones involving only the last two levels in this work, called Early Fusion and Late Fusion.

The first strategy (Early Fusion) deals with the concatenation features vectors ${\left\{X_{a}\right\}}_{s=1}^{N}$ in a single vector X of dimension $d=\overset{N}{\underset{s=1}{\sum }}d_{s}$. The second strategy (Late Fusion) is based on the Late Fusion using Average Probability (LFA) method. The LFA uses a simple heuristic based on the average probabilities of several ensembles results. The LFA guarantees equal weights for each sensor in an attempt to eliminate the influence of irrelevant sensors. The third strategy is based on the Late Fusion using Learned Weights (LFL) method. The LFL considers the weights of each sensor to make the final decision, as there are some sensors that recognize some activities better than others.

Although Vaizman et al. [135] have proposed strategies based on LFA and LFL, most of the work applies the first strategy based on the features’ concatenation. Thus, to complement this strategy, some solutions use dimensionality reduction algorithms such as PCA, LDA, and KDA to aid in the data fusion process. Basically, these algorithms are used in the final step of the data fusion process to improve the decision boundary between the classes composed by the set of concatenated features. The main works that have developed solutions using data fusion are presented below.

Shoaib et al. [57] developed a solution using data from the inertial sensors (accelerometer, a linear accelerometer, gyroscope, and magnetometer) located at various positions of the user’s body. The solution was applied in three scenarios. The first scenario evaluated the classification models with the smartphone located on only one position of the user’s body (waist). The second scenario evaluated the classification models with the smartphone located at other positions of the body like the arm, wrist, and pants pocket. The third scenario evaluated specific classification models of the same users.

Guiry et al. [51] developed a solution for smartphones and smartwatches using data from the accelerometer, gyroscope, magnetometer, light, pressure, and GPS sensors. The data fusion step was divided into 3 steps. The first one deals with the application of the linear interpolation technique with the purpose of synchronizing the extracted data with different frequencies, since the data of the accelerometer, gyroscope, magnetometer, and pressure were collected at a frequency of 100 Hz, 27 Hz, 25 Hz, and 5 Hz, respectively. The second deals with the concatenation of feature vectors of all data sources. Finally, the third deals with the application of the PCA technique to reduce the data dimensionality.

Khan et al. [31] present a solution for smartphones using the data from the accelerometer, pressure and microphone sensors located in various positions of the user’s body. After the process of the feature concatenation, the data fusion step counts with a detailed analysis of the impact of the dimensionality reduction methods PCA, LDA, and KDA in the accuracy of the classification models. The analysis concluded that the KDA method with the RBF (Gaussian) kernel gets the best results.

Other works, such as Vepakomma et al. [128], combine data from inertial sensors with smart environment sensors. All the work cited above was developed using conventional feature extraction methods. In the context of deep learning, some works [118,136] combined data from the inertial accelerometer and gyroscope sensors in the neural network architecture itself without any extra preprocessing with respect to data fusion.

In the hardware context, data fusion techniques are also being implemented in the sensing units themselves. Bancroft and Lachapelle [137], for example, proposed a data fusion solution for multiple IMU (Inertial Measurement Unit) chips. In addition, modern smartphones have IMU chips with built-in data fusion implementations. Thus, the signal extractions do not require any preprocessing, such as orientation, gravity, and linear acceleration. At this point, the time and frequency features can be directly calculated.

## 7. Energy Efficiency

An important aspect that has not been addressed in previous surveys is the presentation of research that is concerned with the development of solutions based on low power consumption of mobile devices. In this perspective, we present recent solutions, based on the data analysis of inertial sensors, related to saving energy of smartphone batteries.

The excessive battery power consumption of smartphones by HAR solutions is still considered one of the main problems preventing the massive spread of applications. The problem is related to the high data processing load which still consumes quite a bit of computational resources. In addition, data collection through the continuous use of sensors also contributes to high power consumption. To solve these problems, the literature presents three strategies to minimize the battery power consumption of smartphones. All of them make a tradeoff between energy consumption and the accuracy of classification models.

The first strategy is related to the selection and activation of on demand sensors, i.e., each sensor is only used when the data is needed to recognize a certain activity [15,45,138]. From the perspective of inertial sensors, the intelligent use of sensors extends to select only a few coordinates of the axes x, y, and z. For example, Viet et al. [139] only use the data of the coordinates y and z. The second strategy deals with the use of lightweight features only to reduce the data processing load [139,140,141,142]. For example, Khan et al. [2] concluded that time domain features have lower computational costs and consume less energy when compared to frequency domain features.

The third strategy deals with the dynamic regulation of the data collection frequency of the inertial sensors for different activities. The higher the frequency of data collection, the more energy that is expended by the inertial sensors. Yan et al. [141] performed an extensive study for each activity where data collection frequencies ranged from 5 Hz to 100 Hz. The results showed that the frequency of 5 Hz is enough to represent static activities such as standing and sitting. On the other hand, the more agitated activities, such as walking and running, need data extracted at a higher frequency, and the frequency of 50 Hz is enough to represent these activities.

## 8. Discussion

These approaches have been widely studied in the literature in recent years. In this sense, this section presents a summary of the main results obtained from experiments performed on different databases. In the context of HAR for smartphones, the literature presents two types of databases that are commonly used in the experiments for HAR solutions validation. The first type of database is those generated by the authors themselves and the second type deals with publicly available databases. Early HAR-based smartphone studies used proprietary databases in their experiments. The disadvantage of such scenario is that, in addition to the difficulty of data collection by the authors, comparisons between the solutions were impaired due to the impossibility of reproducing the experiments in the same scenario. Only after 2011, with the publication of the public WISDM database [49], did the comparison between several solutions of HAR became more feasible. Since then, several other public databases have appeared in the literature. Table 11 lists the main databases of inertial sensors used in research work. It is important to point out that there are other databases listed in [19] and [143], but the others are not frequently used or belong to other domains, like wearable or environmental sensors.

The main results about HAR solutions from the perspective of the inertial sensors embedded in smartphones are presented below. The data presented below are based on the main studies that performed comparative experiments between the different methods and strategies presented in this research.

1. In general, the accelerometer is the predominant sensor in the process of recognizing users’ physical activities, except for some isolated activities like ascending and descending stairs where the gyroscope predominates [57].
2. The data extracted from the gyroscope complements the accelerometer data and both generate better classification models with an average increase of 2% in accuracy [21,22,57]. Wang et al. [59] observed that such fusion is most accurate for the recognition of static activities (e.g., standing and sitting) than for dynamic activities (e.g., walking and running).
3. Accelerometer data without gravity information (linear accelerometer) generates models with less accuracy compared to the accelerometer data with gravity information [58].
4. The magnetometer sensor, when used alone, generates classification models with low accuracy compared to the accelerometer and gyroscope sensors [57].
5. The ideal size for the fixed time windows varies around 2 to 5 s considering a frequency of 20 Hz to 50 Hz [21,53,58].
6. The waist and trouser pocket is the best position to recognize simple physical activities, such as walking and running, since human body movements originate in these regions [44].
7. Time domain features, especially the mean, variance, standard deviation, Root Mean Square, minimum, maximum, amplitude, and correlation, generate models with higher accuracy compared to the characteristics of the frequency domain. In addition, the time features are cheaper and consume less battery power in relation to frequency domain features [49].
8. Wavelet derived features are better than FFT derived features [58].
9. Independent orientation features derived from the magnitude and vertical and horizontal components do not sufficiently represent physical activities [58].
10. KDA is the best dimensionality reduction method when compared to PCA, LDA, and conventional methods of feature selection [31].
11. The RNN method is recommended to recognize activities of short duration and the CNN method is recommended to recognize repetitive activities and long duration [20]. In contrast, the LSTM methods can recognize long-lived activities due to their ability to manipulate multiple memory units [132].
12. Almaslukh et al. [60] proposed an architecture based on the SAE method that generated the best classification model known so far with an accuracy of 97.5%. Such a model overcame the state of the art of conventional methods generated by One-Vs-One Multiclass linear SVM [149] with a 96.4% accuracy.

## 9. Conclusions

This article presents an overview of the HAR area, focusing on smartphones with inertial sensors. We first discussed the concept of human activities followed by a complete history of the HAR area based on smartphones. In this history, the main historical landmarks, representing the evolution of the HAR area over time, have been described. The aim of the history is to help situate the scientific community in the state of the art for the HAR area in the context of smartphones and to present a motivation for the planning and execution of the next steps that will help define the future evolutionary milestones of the area.

In addition, this article presented a detailed description of each step of the methodology commonly used to recognize human activities with smartphones equipped with inertial sensors. In the descriptions of the steps, the main works from the literature, along with tips for the best practices, are presented. In particular, issues related to the features used in classification models were highlighted. In this perspective, we present two approaches to extraction of features based on the way the features are extracted, that is, whether they are manually or automatically extracted.

Such approaches are based on the use of shallow and deep machine learning algorithms. In addition, this paper presented some topics that were not covered in a comprehensive way by the main surveys of the area, as the data fusion, energy efficiency and reduction of data dimensionality topics. Furthermore, we addressed the key findings inherent in the best methods and implementation of HAR based smartphones with inertial sensors.

We presented a set of challenges and future research opportunities in the area of smartphone-based HAR. Additionally, we presented a list of practical applications where HAR solutions can be used in real environments. Therefore, we hope that the information in this article will help the scientific community to create more robust solutions that can increasingly and efficiently recognize users’ physical activities.

## Figures and Tables

**Table 1:** List of works separated by data collection types.

Collection Type	Works
Natural	[25,31,39,43,45,47,52,67,68,69,70,71,72]
Semi-natural	[34,36,49,73,74,75,76]
Laboratory	[4,21,30,37,38,41,42,46,47,50,58,65,67,72,77,78,79,80]

**Table 2:** List of works separated by frequency rate of data collection.

Frequency (Hz)	Works
1–20	[4,36,38,43,45,46,47,49,50,52,69,74,77,78,80]
30–80	[25,30,31,34,45,48,52,58,65,70,71,75,76,77,79,82]
100–200	[39,41,52,72,73,78]
250–16,000	[4,36,38,43,45,46,47,49,50,52,69,74,77,78,80]

**Table 3:** List of works separated by smartphone position on the user’s body.

Position on User’s Body	Works
Any position	[31,36,37,38,39,42,52,65,72,75,80]
Waist	[4,34,46,48,57,58,67,68,76,77,79]
Pants pocket	[21,31,34,41,47,48,49,50,57,67,71,74,76,77,79]
Cord on the neck	[67]
Hand	[21,57,74,79]
Arm	[34,57]
Chest	[31,34,48,76,77]
Backpack	[34,74]

**Table 4:** List of works separated by smartphone orientation on the user’s body.

Orientation	Works
Dependent	[4,21,30,31,34,39,42,45,46,47,49,50,58,67,68,69,78,82]
Independent	[25,36,37,38,41,43,48,58,65,70,71,72,73,74,75,76,77,79,80]

**Table 5:** List of works separated by time window size.

Time Window Size (Seconds)	Works
<1	[31,39,42,47,48,76,78]
1–5	[21,30,31,34,37,41,48,50,52,53,58,70,72,73,74,76,78,79,80,83]
7–60	[21,25,30,36,49,71,75,82]

**Table 6:** Time domain features used in the literature.

Domain	Features
Time	min, max, amplitude, amplitude peak, sum, absolute sum, Euclidian norm, mean, absolute mean, mean square, mean absolute deviation, sum square error, variance, standard deviation, Pearson coefficient, zero crossing rate, correlation, cross-correlation, auto-correlation, skewness, kurtosis, area, absolute area, signal magnitude mean, absolute signal magnitude mean, magnitude difference function.

**Table 7:** Frequency domain features used in the literature.

Domain	Features
Frequency	Energy, energy normalized, power, centroid, entropy, DC component, peak, coefficient sum.

**Table 8:** List of works separated by domain features.

Feature Domain	Works
Time	[4,25,30,31,34,36,38,39,41,43,45,47,49,50,52,65,67,68,69,70,71,72,73,74,75,76,77,78,79,80,82]
Frequency	[25,34,37,41,45,68,70,72,74,79,80,82]

**Table 9:** List of works separated by shallow machine learning algorithms.

Methods	Works
Naïve Bayes	[25,30,36,37,41,45,50,69,74,78,80]
Decision Tree	[25,30,34,42,43,45,49,50,67,70,74,75,79,80]
Support Vector Machine (SVM)	[25,31,36,45,46,50,72,74,82]
KNN	[25,42,48,50,71,73,74,76,78]
Neural Networks	[30,38,49,52]

**Table 10:** List of works separated by shallow machine learning algorithms.

Methods	Works
SAE	[60,93,94]
RBM	[54,95,96,97,98,99,100,101,102]
CNN	[91,94,99,103,104,105,106,107,108,109,110,111,112,113,114,115,116,117,118,119,120,121,122,123,124]
RNN	[90,91,108,119,125,126,127]
DFN	[108,119,128,129,130]
DBN	[131]
LSTM	[132]

**Table 11:** List of public databases. A–accelerometer, G–gyroscope, and M–magnetometer.

Datasets	Frequency	Sensors	Subjects	Nº Class	Reference
OPPORTUNITY	30 Hz	A, G, M	12	15	[144]
UCI-HAR	50 Hz	A, G	30	6	[145]
PAMAP2	100 Hz	A, G, M	9	23	[32]
USC-HAD	100 Hz	A, G	14	12	[146]
WISDM and Actitracker	20 Hz	A	29	7	[49]
MHealth	50 Hz	A, G	10	12	[147]
Extra Sensory	40 Hz	A, G, M	60	51	[135]
Shoaib	50 Hz	A, G, M	10	7	[57]
UniMib Shar	50 Hz	A	30	17	[148]

## References

[1] Lockhart J.W., Pulickal T., Weiss G.M. Applications of mobile activity recognition. Proceedings of the 2012 ACM Conference on Ubiquitous Computing–UbiComp.

[2] Khan W.Z., Xiang Y., Aalsalem M.Y., Arshad Q. (2013). Mobile phone sensing systems: A survey. IEEE Commun. Surv. Tutor..

[3] Dai J., Bai X., Yang Z., Shen Z., Xuan D. PerFallD: A pervasive fall detection system using mobile phones. Proceedings of the 8th IEEE International Conference on Pervasive Computing and Communications Workshops (PERCOM Workshops).

[4] Fontecha J., Navarro F.J., Hervás R., Bravo J. (2013). Elderly frailty detection by using accelerometer-enabled smartphones and clinical information records. Pers. Ubiquitous Comput..

[5] Preuveneers D., Berbers Y. Mobile phones assisting with health self-care: A diabetes case study. Proceedings of the 10th International Conference on Human Computer Interaction with Mobile Devices and Services.

[6] Tapia E.M., Intille S.S., Larson K. (2004). Activity recognition in the home using simple and ubiquitous sensors. International Conference on Pervasive Computing.

[7] Lima W.S., Souto E., Rocha T., Pazzi R.W., Pramudianto F. User activity recognition for energy saving in smart home environment. Proceedings of the IEEE Symposium on Computers and Communication (ISCC).

[8] Niu W., Long J., Han D., Wang Y.F. Human activity detection and recognition for video surveillance. Proceedings of the IEEE International Conference on Multimedia and Exp (ICME).

[9] Mehran R., Oyama A., Shah M. Abnormal crowd behavior detection using social force model. Proceedings of the 2009 IEEE Conference on Computer Vision and Pattern Recognition.

[10] Viola P., Jones M. Rapid object detection using a boosted cascade of simple features. Proceedings of the IEEE Computer Society Conference on Computer Vision and Pattern Recognition (CVPR).

[11] Choujaa D., Dulay N. (2009). Activity Recognition from Mobile Phone Data: State of the Art, Prospects and Open Problems. Imp. Coll. Lond..

[12] Liao L., Patterson D.J., Fox D., Kautz H. (2007). Learning and inferring transportation routines. Artif. Intell..

[13] Saeedi S. (2013). Context-Aware Personal Navigation Services Using Multi-Level Sensor Fusion Algorithms. Ph.D. Thesis.

[14] Chen L., Hoey J., Nugent C.D., Cook D.J., Yu Z., Member S. (2012). Sensor-Based Activity Recognition. Syst. Man Cybern. Part C Appl. Rev..

[15] Lane N.D., Miluzzo E., Lu H., Peebles D., Choudhury T., Campbell A.T. (2010). A survey of mobile phone sensing. IEEE Commun. Mag..

[16] Incel O.D., Kose M., Ersoy C. (2013). A Review and Taxonomy of Activity Recognition on Mobile Phones. BioNanoScience.

[17] bin Abdullah M.F.A., Ali F.P.N., Sayeed M.S., Choi D.J., Muthu K.S. (2012). Classification algorithms in human activity recognition using smartphones. Int. J. Med Health Biomed. Bioeng. Pharm. Eng..

[18] Khusainov R., Azzi D., Achumba I.E., Bersch S.D. (2013). Real-time human ambulation, activity, and physiological monitoring: Taxonomy of issues, techniques, applications, challenges and limitations. Sensors.

[19] Wang J., Chen Y., Hao S., Peng X., Hu L. (2017). Deep Learning for Sensor-based Activity Recognition: A Survey. Comput. Vis. Pattern Recognit..

[20] Cook D.J., Krishnan C.N. (2015). Activity Learning: Discovering, Recognizing, and Predicting Human Behavior from Sensor Data.

[21] Shoaib M., Bosch S., Incel O.D., Scholten H., Havinga P.J. (2016). Complex human activity recognition using smartphone and wrist-worn motion sensors. Sensors.

[22] Chen Y., Shen C. (2017). Performance Analysis of Smartphone-Sensor Behavior for Human Activity Recognition. IEEE Access.

[23] Lara O.D., Labrador M.A. (2013). A Survey on Human Activity Recognition using Wearable Sensors. IEEE Commun. Surv. Tutor..

[24] Bulling A., Blanke U., Schiele B. (2014). A tutorial on human activity recognition using body-worn inertial sensors. ACM Comput. Surv..

[25] Yang J. Toward Physical Activity Diary: Motion Recognition Using Simple Acceleration Features with Mobile Phones. Proceedings of the 1st International Workshop on Interactive Multimedia for Consumer Electronics.

[26] Avci A., Bosch S. Activity recognition using inertial sensing for healthcare, wellbeing and sports applications: A survey. Proceedings of the 23th International conference on architecture of computing systems (ARCS).

[27] Su X., Tong H., Ji P. (2014). Activity recognition with smartphone sensors. Sci. Technol..

[28] Bort-Roig J., Gilson N.D., Puig-Ribera A., Contreras R.S., Trost S.G. (2014). Measuring and influencing physical activity with smartphone technology: A systematic review. Sports Med..

[29] Li F., Shirahama K., Nisar M., Köping L., Grzegorzek M. (2018). Comparison of Feature Learning Methods for Human Activity Recognition Using Wearable Sensors. Sensors.

[30] Dernbach S., Das B., Krishnan N.C., Thomas B.L., Cook D.J. Simple and Complex Activity Recognition through Smart Phones. Proceedings of the 2012 Eighth International Conference on Intelligent Environments.

[31] Khan A.M., Tufail A., Khattak A.M., Khattak A.M., Laine T.H. (2014). Activity recognition on smartphones via sensor-fusion and KDA-based SVMs. Int. J. Distrib. Sens. Netw..

[32] Reiss A., Weber M., Stricker D. Exploring and extending the boundaries of physical activity recognition. Proceedings of the 2011 IEEE International Conference on Systems, Man, and Cybernetics.

[33] Li M., Zhou P., Zheng Y., Li Z., Shen G. (2014). IODetector: A Generic Service for Indoor/Outdoor Detection. ACM Trans. Sens. Netw..

[34] Reddy S., Mun M., Burke J., Estrin D., Hansen M., Srivastava M. (2010). Using mobile phones to determine transportation modes. ACM Trans. Sens. Netw..

[35] Wohlin C. Guidelines for snowballing in systematic literature studies and a replication in software engineering. Proceedings of the 18th International Conference on Evaluation and Assessment in Software Engineering.

[36] Sohn T., Varshavsky A., Lamarca A., Chen M.Y., Choudhury T., Smith I., Consolvo S., Hightower J., Griswold W.G., Lara E.D. (2006). Mobility Detection Using Everyday GSM Traces. International Conference on Ubiquitous Computing.

[37] Iso T., Yamazaki K. Gait analyzer based on a cell phone with a single three-axis accelerometer. Proceedings of the 8th Conference on Human-Computer Interaction with Mobile Devices and Services.

[38] Anderson I., Maitland J., Sherwood S., Barkhuus L., Chalmers M., Hall M., Brown B., Muller H. (2007). Shakra: Tracking and sharing daily activity levels with unaugmented mobile phones. Mob. Netw. Appl..

[39] Berchtold M., Budde M., Gordon D., Schmidtke H., Beigl M. ActiServ: Activity Recognition Service for mobile phones. Proceedings of the International Symposium on Wearable Computers (ISWC).

[40] Miluzzo E., Cornelius C.T., Ramaswamy A., Choudhury T., Liu Z., Campbell A.T. Darwin Phones: The Evolution of Sensing and Inference on Mobile Phones. Proceedings of the 8th International Conference on Mobile Systems, Applications, and Services.

[41] Saponas T., Lester J., Froehlich J., Fogarty J., Landay J. (2008). iLearn on the iPhone: Real-Time Human Activity Classification on Commodity Mobile Phones.

[42] Lu H., Pan W., Lane N., Choudhury T., Campbell A. SoundSense: Scalable sound sensing for people-centric applications on mobile phones. Proceedings of the 7th International Conference on Mobile Systems, Applications, and Services.

[43] Ryder J., Longstaff B., Reddy S., Estrin D. Ambulation: A Tool for Monitoring Mobility Patterns over Time Using Mobile Phones. Proceedings of the International Conference on Computational Science and Engineering.

[44] Purpura S., Schwanda V., Williams K., Stubler W., Sengers P. Fit4life: The design of a persuasive technology promoting healthy behavior and ideal weight. Proceedings of the 2011 Annual Conference on Human Factors in Computing Systems–CHI.

[45] Lu H., Yang J., Liu Z., Lane N.D., Choudhury T., Campbell A.T. The Jigsaw Continuous Sensing Engine for Mobile Phone Applications. Proceedings of the 8th Conference on Embedded Networked Sensor Systems (SenSys’10).

[46] Zhang S., McCullagh P., Nugent C., Zheng H. Activity Monitoring Using a Smart Phone’s Accelerometer with Hierarchical Classification. Proceedings of the 2010 Sixth International Conference on Intelligent Environments.

[47] Bieber G., Koldrack P., Sablowski C., Peter C., Urban B. Mobile physical activity recognition of stand-up and sit-down transitions for user behavior analysis. Proceedings of the 3rd International Conference on PErvasive Technologies Related to Assistive Environments.

[48] Henpraserttae A., Thiemjarus S., Marukatat S. Accurate activity recognition using a mobile phone regardless of device orientation and location. Proceedings of the 2011 International Conference on Body Sensor Networks BSN.

[49] Kwapisz J.R., Weiss G.M., Moore S.A. (2011). Activity Recognition using Cell Phone Accelerometers. ACM SIGKDD Explor. Newsl..

[50] Das B., Seelye A.M., Thomas B.L., Cook D.J., Holder L.B., Schmitter-Edgecombe M. Using smart phones for context-aware prompting in smart environments. Proceedings of the IEEE Consumer Communications and Networking Conference, CCNC.

[51] Guiry J.J., van de Ven P., Nelson J. (2014). Multi-sensor fusion for enhanced contextual awareness of everyday activities with ubiquitous devices. Sensors.

[52] Khan A.M., Siddiqi M.H., Lee S.W. (2013). Exploratory data analysis of acceleration signals to select light-weight and accurate features for real-time activity recognition on smartphones. Sensors.

[53] Banos O., Galvez J.M., Damas M., Pomares H., Rojas I. (2014). Window Size Impact in Human Activity Recognition. Sensors.

[54] Wang J., Chen Y., Hao S., Peng X., Hu L. Deep Activity Recognition Models with Triaxial Accelerometers. Proceedings of the Computer Vision and Pattern Recognition.

[55] Abdallah Z.S., Gaber M.M., Srinivasan B., Krishnaswamy S. (2016). AnyNovel: Detection of novel concepts in evolving data streams. Evol. Syst..

[56] Reyes-Ortiz J.L., Oneto L., Samà A., Parra X., Anguita D. (2016). Transition-Aware Human Activity Recognition Using Smartphones. Neurocomputing.

[57] Shoaib M., Bosch S., Incel O.D., Scholten H., Havinga P.J. (2014). Fusion of smartphone motion sensors for physical activity recognition. Sensors.

[58] Sousa W., Souto E., Rodrigres J., Sadarc P., Jalali R., El-khatib K. A Comparative Analysis of the Impact of Features on Human Activity Recognition with Smartphone Sensors. Proceedings of the 23rd Brazillian Symposium on Multimedia and the Web.

[59] Wang A., Chen G., Yang J., Zhao S., Chang C.Y. (2016). A Comparative study on Human activity recognition using inertial sensors in a smartphone. IEEE Sens. J..

[60] Almaslukh B., Almuhtadi J., Artoli A. (2017). An Effective Deep Autoencoder Approach for Online Smartphone-Based Human Activity Recognition. Int. J. Comput. Sci. Netw. Secur..

[61] Köping L., Shirahama K., Grzegorzek M. (2018). A General Framework for Sensor-based Human Activity Recognition. Comput. Biol. Med..

[62] Bengio Y. (2013). Deep learning of representations: Looking forward. International Conference on Statistical Language and Speech Processing.

[63] Figo D., Diniz P.C., Ferreira D.R., Cardoso J.M.P. (2010). Preprocessing techniques for context recognition from accelerometer data. Pers. Ubiquitous Comput..

[64] LeCun Y., Bengio Y., Hinton G. (2015). Deep learning. Nature.

[65] Mladenov M., Mock M. A step counter service for Java-enabled devices using a built-in accelerometer. Proceedings of the 1st International Workshop on Context-Aware Middleware and Services affiliated with the 4th International Conference on Communication System Software and Middleware (COMSWARE 2009).

[66] Lockhart J.W., Weiss G.M. Limitations with Activity Recognition Methodology & Data Sets. Proceedings of the 2014 ACM International Joint Conference on Pervasive and Ubiquitous Computing: Adjunct Publication.

[67] Miluzzo E., Lane N.D., Fodor K., Peterson R., Lu H., Musolesi M., Eisenman S.B., Zheng X., Campbell A.T. Sensing Meets Mobile Social Networks: The Design, Implementation and Evaluation of the CenceMe Application. Proceedings of the 6th ACM Conference on Embedded Network Sensor Systems.

[68] Lane N., Mohammod M., Lin M., Yang X., Lu H., Ali S., Doryab A., Berke E., Choudhury T., Campbell A. (2011). BeWell: A Smartphone Application to Monitor, Model and Promote Wellbeing. Proceedings of the 5th International ICST Conference on Pervasive Computing Technologies for Healthcare.

[69] Gomes J.B., Krishnaswamy S., Gaber M.M., Sousa P.A., Menasalvas E. MARS: A personalised mobile activity recognition system. Proceedings of the 2012 IEEE 13th International Conference on Mobile Data Management MDM.

[70] Lara O.D., Labrador M.A. A mobile platform for real-time human activity recognition. Proceedings of the Consumer Communications and Networking Conference (CCNC).

[71] Siirtola P., Röning J. (2012). Recognizing Human Activities User-independently on Smartphones Based on Accelerometer Data. Int. J. Interact. Multimed. Artif. Intell..

[72] Park J.G., Patel A., Curtis D., Teller S., Ledlie J. Online pose classification and walking speed estimation using handheld devices. Proceedings of the 2012 ACM Conference on Ubiquitous Computing–UbiComp.

[73] Ustev Y., Incel O.D., Ersoy C. User, device and orientation independent human activity recognition on mobile phones: Challenges and a proposal. Proceedings of the ACM Conference on Pervasive and Ubiquitous Computing Adjunct Publication.

[74] Anjum A., Ilyas M.U. Activity recognition using smartphone sensors. Proceedings of the IEEE 10th Consumer Communications and Networking Conference, CCNC.

[75] Siirtola P., Roning J. Ready to use activity recognition for smartphones. Proceedings of the IEEE Symposium on Computational Intelligence and Data Mining, CIDM.

[76] Thiemjarus S., Henpraserttae A., Marukatat S. A study on instance-based learning with reduced training prototypes for device-context-independent activity recognition on a mobile phone. Proceedings of the 2013 IEEE International Conference on Body Sensor Networks, BSN.

[77] Hynes M., Wang H., McCarrick E., Kilmartin L. (2011). Accurate monitoring of human physical activity levels for medical diagnosis and monitoring using off-the-shelf cellular handsets. Pers. Ubiquitous Comput..

[78] Kose M., Incel O.D., Ersoy C. Online Human Activity Recognition on Smart Phones. Proceedings of the 2nd International Workshop on Mobile Sensing: From Smartphones and Wearables to Big Data.

[79] Schindhelm C.K. Activity recognition and step detection with smartphones: Towards terminal based indoor positioning system. Proceedings of the IEEE International Symposium on Personal, Indoor and Mobile Radio Communications, PIMRC.

[80] Martín H., Bernardos A.M., Iglesias J., Casar J.R. (2013). Activity logging using lightweight classification techniques in mobile devices. Pers. Ubiquitous Comput..

[81] Shoaib M., Bosch S., Incel O., Scholten H., Havinga P. (2015). A Survey of Online Activity Recognition Using Mobile Phones. Sensors.

[82] Vo Q.V., Hoang M.T., Choi D. (2013). Personalization in mobile activity recognition system using-medoids clustering algorithm. Int. J. Distrib. Sens. Netw..

[83] Yan Z., Misra A., Chakraborty D., Aberer K., Jeung H. (2012). Semantic Activity Classification Using Locomotive Signatures from Mobile Phones.

[84] Sebastião R., Silva M.M., Rabiço R., Gama J., Mendonça T. (2013). Evolving Systems. Real-time algorithm for changes detection in depth of anesthesia signals. Evolving Syst..

[85] Strang G. (1994). Wavelets. Sigma Xi. Sci. Res. Soc..

[86] Chu D., Lane N.D., Lai T.T.T., Pang C., Meng X., Guo Q., Li F., Zhao F. Balancing energy, latency and accuracy for mobile sensor data classification. Proceedings of the 9th ACM Conference on Embedded Networked Sensor Systems–SenSys.

[87] Lee J., Verleysen M. (2007). Nonlinear Dimensionality Reduction.

[88] Khan A.M. (2011). Human Activity Recognition Using A Single Tri-axial Accelerometer. Ph.D. Thesis.

[89] Goodfellow I., Bengio Y., Courville A. (2016). Deep Learning.

[90] Ordónez F., Roggen D. (2016). Deep convolutional and LSTM recurrent neural networks for multimodal wearable activity recognition. Sensors.

[91] Yao S., Hu S., Zhao Y., Zhang A., Abdelzaher T. Deepsense: A unified deep learning framework for time-series mobile sensing data processing. Proceedings of the 26th International Conference on World Wide Web, International WWW Conferences Steering Committee.

[92] Garcia C.E., Brena R.F. (2016). Activity recognition using community data to complement small amounts of labeled instances. Sensors.

[93] Li Y., Shi D., Ding B., Liu D. (2014). Unsupervised feature learning for human activity recognition using smartphone sensors. Mining Intelligence and Knowledge Exploration.

[94] Zheng Y., Liu Q., Chen E., Ge Y., Zhao J. (2014). Time series classification using multi-channels deep convolutional neural networks. Conference on Web-Age Information Management.

[95] Bhattacharya S., Lane N. From smart to deep: Robust activity recognition on smartwatches using deep learning. Proceedings of the 2016 IEEE International Conference on Pervasive Computing and Communication Workshops.

[96] Hammerla N., Fisher J., Andras P., Rochester L., Walker R. Pd disease state assessment in naturalistic environments using deep learning. Proceedings of the Twenty-Ninth AAAI Conference on Artificial Intelligence.

[97] Hayashi T., Nishida M., Kitaoka N., Takeda K. Daily activity recognition based on dnn using environmental sound and acceleration signals. Proceedings of the 2015 23rd European Signal Processing Conference (EUSIPCO).

[98] Lane N., Georgiev P. Can deep learning revolutionize mobile sensing? In Proceedings of the 16th International Workshop on Mobile Computing Systems and Applications.

[99] Liu C., Zhang L., Liu Z., Liu K., Li X., Liu Y. Lasagna: Towards deep hierarchical understanding and searching over mobile sensing data. Proceedings of the 22nd Annual International Conference on Mobile Computing and Networking.

[100] Plötz T., Hammerla N.Y., Olivier P.L. Feature learning for activity recognition in ubiquitous computing. Proceedings of the International Joint Conference on Artificial Intelligence.

[101] Radu V., Lane N., Bhattacharya S., Mascolo C., Marina M., Kawsar F. Towards multimodal deep learning for activity recognition on mobile devices. Proceedings of the 2016 ACM International Joint Conference on Pervasive and Ubiquitous Computing: Adjunct.

[102] Zhang L., Wu X., Luo D. Real-time activity recognition on smart-phones using deep neural networks. Proceedings of the 2015 IEEE 12th Intl Conference on Ubiquitous Intelligence and Computing and 2015 IEEE 12th Intl Conference on Autonomic and Trusted Computing and 2015 IEEE 15th Intl Conference on Scalable Computing and Communications and Its Associated Workshops (UIC-ATC-ScalCom).

[103] Chen Y., Xue Y. A deep learning approach to human activity recognition based on single accelerometer. Proceedings of the 2015 IEEE International Conference on Systems, Man, and Cybernetics.

[104] Chen Y., Zhong K., Zhang J., Sun Q., Zhao X. LSTM networks for mobile human activity recognition. Proceedings of the 2016 International Conference on Artificial Intelligence: Technologies and Applications.

[105] Gjoreski H., Bizjak J., Gjoreski M., Gams M. Comparing deep and classical machine learning methods for human activity recognition using wrist accelerometer. Proceedings of the IJCAI 2016 Workshop on Deep Learning for Artificial Intelligence.

[106] Ha S., Yun J., Choi S. Multi-modal convolutional neural networks for activity recognition. Proceedings of the 2015 IEEE International Conference on Systems, Man, and Cybernetics.

[107] Ha S., Choi S. Convolutional Neural Networks for human activity recognition using multiple accelerometer and gyroscope sensors. Proceedings of the 2016 International Joint Conference on Neural Networks (IJCNN).

[108] Hammerla N., Halloran S., Ploetz T. (2016). Deep, convolutional, and recurrent models for human activity recognition using wearables. arXiv.

[109] Hannink J., Kautz T., Pasluosta C., Gabmann K., Klucken J., Eskofier B. (2017). Sensor-based gait parameter extraction with deep convolutional neural networks. IEEE J. Biomed. Health Inform..

[110] Jiang W., Yin Z. Human activity recognition using wearable sensors by deep convolutional neural networks. Proceedings of the 23rd ACM international conference on Multimedia.

[111] Kim Y., Li Y. (2017). Human activity classification with transmission and reflection coefficients of on-body antennas through deep convolutional neural networks. IEEE Trans. Antennas Propag..

[112] Lee S., Yoon S., Cho H. Human activity recognition from accelerometer data using convolutional neural network. Proceedings of the 2017 IEEE International Conference on Big Data and Smart Computing (BigComp).

[113] Mohammed S., Tashev I. Unsupervised deep representation learning to remove motion artifacts in free-mode body sensor networks. Proceedings of the 2017 IEEE 14th International Conference on Wearable and Implantable Body Sensor Networks (BSN).

[114] Morales F., Roggen D. Deep convolutional feature transfer across mobile activity recognition domains, sensor modalities and locations. Proceedings of the 2016 ACM International Symposium on Wearable Computers.

[115] Pourbabaee B., Roshtkhari M., Khorasani K. (2017). Deep convolution neural networks and learning ecg features for screening paroxysmal atrial fibrillatio patients. IEEE Trans. Syst. Man Cybern Syst..

[116] Ravi D., Wong C., Lo B., Yang G. Deep learning for human activity recognition: A resource efficient implementation on low-power devices. Proceedings of the 2016 IEEE 13th International Conference on Wearable and Implantable Body Sensor Networks (BSN).

[117] Ravı D., Wong C., Lo B., Yang G. (2017). A deep learning approach to on-node sensor data analytics for mobile or wearable devices. IEEE J. Biomed. Health Inform..

[118] Ronao C., Cho S. (2015). 2015 Deep convolutional neural networks for human activity recognition with smartphone sensors. International Conference on Neural Information Processing.

[119] Sathyanarayana A., Joty S., Fernandez-Luque L., Ofli F., Srivastava J., Elmagarmid A., Taheri S., Arora T. (2016). Impact of physical activity on sleep: A deep learning based exploration. arXiv.

[120] Wang J., Zhang X., Gao Q., Yue H., Wang H. (2016). Device-free wireless localization and activity recognition: A deep learning approach. IEEE Trans. Veh. Technol..

[121] Yang J., Nguyen M., San P., Li X., Krishnaswamy S. Deep convolutional neural networks on multichannel time series for human activity recognition. Proceedings of the 24th International Joint Conference on Artificial Intelligence (IJCAI).

[122] Zebin T., Scully P., Ozanyan K. Human activity recognition with inertial sensors using a deep learning approach. Proceedings of the 2016 IEEE SENSORS.

[123] Zeng M., Nguyen L., Yu B., Mengshoel O., Zhu J., Wu P., Zhang J. Convolutional Neural Networks for human activity recognition using mobile sensors. Proceedings of the 6th International Conference on Mobile Computing, Applications and Services.

[124] Zheng Y., Liu Q., Chen E., Ge Y., Zhao J. (2016). Exploiting multi-channels deep convolutional neural networks for multivariate time series classification. Front. Comput. Sci..

[125] Edel M. Koppe Binarized-blstm-rnn based human activity recognition. Proceedings of the 2016 International Conference on Indoor Positioning and Indoor Navigation (IPIN).

[126] Guan Y., Ploetz T. (2017). Ensembles of deep LSTM learners for activity recognition using wearables. arXiv.

[127] Inoue M., Inoue S., Nishida T. (2016). Deep recurrent neural network for mobile human activity recognition with high throughput. arXiv.

[128] Vepakomma P., De D., Das S., Bhansali S. A-wristocracy: Deep learning on wrist-worn sensing for recognition of user complex activities. Proceedings of the 2015 IEEE 12th International Conference on Wearable and Implantable Body Sensor Networks (BSN).

[129] Walse K., Dharaskar R., Thakare V. (2016). PCA based optimal ann classifiers for human ACTI Ensembles of deep ITY recognition using mobile sensors data. Proceedings of the First International Conference on Information and Communication Technology for Intelligent Systems.

[130] Zhang L., Wu X., Luo D. Human activity recognition with hmm-dnn model. Proceedings of the 2015 IEEE 14th International Conference on Cognitive Informatics & Cognitive Computing (ICCI* CC).

[131] Zhang L., Wu X., Luo D. Recognizing human activities from raw accelerometer data using deep neural networks. Proceedings of the 2015 IEEE 14th International Conference on Machine Learning and Applications (ICMLA).

[132] Chen Z., Zhang L., Cao Z., Guo J. (2018). Distilling the Knowledge from Handcrafted Features for Human Activity Recognition. IEEE Trans. Ind. Inform..

[133] Klein L.A. (2004). Sensor and Data Fusion: A Tool for Information Assessment and Decision Making.

[134] Tsinganos P., Skodras A. (2018). On the Comparison of Wearable Sensor Data Fusion to a Single Sensor Machine Learning Technique in Fall Detection. Sensors.

[135] Vaizman Y., Ellis K., Lanckriet G. (2017). Recognizing Detailed Human Context In-the-Wild from Smartphones and Smartwatches. IEEE Pervasive Comput..

[136] Hassan M.M., Uddin M.Z., Mohamed A., Almogren A. (2017). A robust human activity recognition system using smartphone sensors and deep learning. Future Gener. Comput. Syst..

[137] Bancroft J.B., Lachapelle G. (2011). Data fusion algorithms for multiple inertial measurement units. Sensors.

[138] Wang Y., Lin J., Annavaram M., Jacobson Q.A., Hong J., Krishnamachari B. A Framework of energy efficient mobile sensing for automatic user state recognition. Proceedings of the 7th International Conference on Mobile Systems, Applications, and Services.

[139] Viet V.Q., Thang H.M., Choi D. Balancing precision and battery drain in activity recognition on mobile phone. Proceedings of the 18th International Conference on Parallel and Distributed Systems–ICPADS.

[140] Liang Y., Zhou X., Yu Z., Guo B. (2014). Energy-efficient motion related activity recognition on mobile devices for pervasive healthcare. Mob. Netw. Appl..

[141] Yan Z., Subbaraju V., Chakraborty D., Misra A., Aberer K. Energy-efficient continuous activity recognition on mobile phones: An activity-adaptive approach. Proceedings of the 2012 16th International Symposium on Wearable Computers.

[142] Viet V.Q., Thang H.M., Choi D. Adaptive energy-saving strategy for activity recognition on mobile phone. Proceedings of the 2012 IEEE International Symposium on Signal Processing and Information Technology (ISSPIT).

[143] Ramamurthy S., Roy N. (2018). Recent trends in machine learning for human activity recognition—A survey. Wiley Interdiscip. Rev. Data Min. Knowl. Discovery.

[144] Chavarriaga R., Sagha H., Calatroni A., Digumarti S.T., Tröster G., Millán J.D.R., Roggen D. (2013). The Opportunity challenge: A benchmark database for on-body sensor-based activity recognition. Pattern Recognit. Lett..

[145] Anguita D., Ghio A., Oneto L., Parra X., Reyes-Ortiz J.L. A Public Domain Dataset for Human Activity Recognition Using Smartphones. Proceedings of the European Symposium on Artificial Neural Networks, Computational Intelligence and Machine Learning.

[146] Zhang M., Sawchuk A.A. USC-HAD: A Daily Activity Dataset for Ubiquitous Activity Recognition Using Wearable Sensors. Proceedings of the 2012 ACM Conference on Ubiquitous Computing.

[147] Banos O., Garcia R., Holgado J.A., Damas M., Pomares H., Rojas I., Saez A., Villalonga C. mHealthDroid: A novel framework for agile development of mobile health applications. Proceedings of the 6th International Work-Conference on Ambient Assisted Living an Active Ageing (IWAAL 2014).

[148] Micucci D., Mobilio M., Napoletano P. (2017). UniMiB SHAR: A new dataset for human activity recognition using acceleration data from smartphones. Appl. Sci..

[149] Romera P.B., Aung M.S., Bianchi-Berthouze N. A one-vs-one classifier ensemble with majority voting for activity recognition. Proceedings of the European Symposium on Artificial Neural Networks, Computational Intelligence and Machine Learning.

